# Comorbidity of sickle cell trait and albinism: a cross-sectional survey in the Democratic Republic of the Congo

**DOI:** 10.11604/pamj.2020.35.127.21113

**Published:** 2020-04-17

**Authors:** Paul Kambale-Kombi, Roland Marini Djang'eing'a, Jean-Pierre Alworong'a Opara, Gaylor Inena wa Inena, Daddy Falay Sadiki, François Boemer, Vincent Bours, Charles Kayembe Tshilumba, Salomon Batina-Agasa

**Affiliations:** 1 Département de Médecine Interne, Cliniques Universitaires de Kisangani, Faculté de Médecine et de Pharmacie, Université de Kisangani, Kisangani, République Démocratique du Congo; 2 Département de Pharmacie, Chimie Analytique Pharmaceutique, CHU de Liège, Faculté de Médecine, Université de Liège, Liège, Belgique; 3 Département de Pédiatrie, Cliniques Universitaires de Kisangani, Faculté de Médecine et de Pharmacie, Université de Kisangani, Kisangani, République Démocratique du Congo; 4 Hôpital du Cinquantenaire de Kisangani, Kisangani, République Démocratique du Congo; 5 Département de Génétique Humaine, CHU de Liège, Faculté de Médecine, Université de Liège, Liège, Belgique

**Keywords:** Comorbidity, sickle cell trait, albinism, Democratic Republic of the Congo

## Abstract

**Introduction:**

Sickle Cell Disease (SCD) and albinism are both recessive hereditary diseases in human kind with a high prevalence in sub-Saharan Africa. This study aimed to determinate the prevalence of sickle cell trait in people living with albinism (PLA).

**Methods:**

a cross-sectional descriptive survey was conducted in PLA attending the "Hôpital du Cinquantenaire de Kisangani". In total, by non-probabilistic convenience sampling, 82 albinos and 139 non-albinos and without any antecedents of albinism in their family were included, selected from students in the Faculty of Medicine and Pharmacy at the University of Kisangani. Blood samples were collected on "dried blood spot" and analyzed by mass spectrometry at CHU of Liège. Data were entered into an Excel file and analysed on SPSS 20.0 (Chicago, IL).

**Results:**

forty-six of the 82 albinos (56.1%) were female and 43.9% male with a sex ratio of 1.28. Among albinos, 18.3% had hemoglobin AS (HbAS) and 81.7% hemoglobin AA (HbAA) compared to 18% of subjects with hemoglobin AS and 82% hemoglobin AA in the control group. The difference was not statistically significant (Chi-square=0.003, ddl=1, p=0.9544).

**Conclusion:**

this study highlighted that the prevalence of the sickle cell trait is high among people living with albinism, but does not differ from that observed in non-albinos in the Democratic Republic of the Congo. It is therefore important to raise awareness among this category of people about sickle cell disease and the importance of its premarital screening.

## Introduction

Sickle cell anemia and albinism are both recessive hereditary diseases in humans with a high prevalence in sub-Saharan Africa populations [1]. Today´s, sickle cell disease (SCD) is the most common genetic disease worldwide. The annual number of newborns with sickle cell disease was estimated to be 305 800 (CI: 238 400-398 800) in 2010 with 79% occurring in sub-Saharan Africa [2]. SCD is an autosomal recessive genetic disease, except in rare forms such as HbSAntilles and HbSOman, due to a mutation at the 6^th^ codon of the β-globin gene that leads to the replacement of adenine by thymine (GAG→GTG). As a result, glutamic acid is replaced by valine at the sixth position of β-globin on chromosome 11, leading to the formation of sickle red blood cells (RBC) containing abnormal hemoglobin (HbS: α2βS2), rather than normal adult hemoglobin (HbA: α2β2) [3].

The polymerization of this abnormal hemoglobin (HbS) under certain conditions such as deoxygenated conditions, makes the sickle red blood cell fragile and less deformable, resulting in the clinical features of the disease. Albinism includes a heterogeneous group of genetic abnormalities that affect 1 in 20,000 individuals worldwide [4]. It is characterized by a reduced or lack of melanin production leading to hypopigmentation of the skin, hair and eyes. Generally, there are three forms of albinism: ocular albinism (OA) which is a X-linked inherited disease related to gene GPR 143, oculocutaneous albinism (OCA) which is an autosomal recessive inherited disease and syndromic forms [5]. Seven subtypes of oculocutaneous albinism have been described. However, four subtypes are better characterized and the genes involved are: TYR on chromosome 11q14 for OCA 1, OCA 2 (P) for OCA 2, TYRP1 for OCA 3 and SLC45A2 (MATP) for OCA 4. The syndromic forms include Hermansky-Pudlak syndrome, related to the HPS-1 gene and Chediak-Higashi syndrome, related to the CHS-1 gene [4,6-11].

It has been estimated that albinism affects 1 in 20000 people worldwide; in Africa, incidences range from 1 in 2700 to 1 in 10000 people [6]. Oculocutaneous albinism is the most common type in sub-Saharan Africa with estimated prevalences of 1 in 1755 in Namibia, 1 in 2675 in Tanzania, 1 in 4728 in Zimbabwe and 1 in 3900 in South Africa. The overall prevalence of albinism in sub-Saharan Africa is estimated at 1 in 2000-5000 [7,8,12,13]. Oculocutaneous albinism, which is an autosomal recessive hereditary disease has been documented to co-exist with abnormal hemoglobin conditions like sickle cell disease [1]. But surveys that have focused on this co-morbidity are scarce in sub-Saharan Africa and the two studies that reported this observation, each involved only one albino family [14,15]. In the DRC, albinism is known to be widespread, nevertheless there have been no prevalence studies. Similarly, the prevalence of sickle cell disease is high, estimated at about 1.4% for the homozygous form [16] and 23.3% for the heterozygous form [17].

In addition, WHO estimates that the AS carrier rate is 25% and the annual incidence of the homozygous SS form around 15‰ births in the DRC [18], which is one of the three most affected countries by SCD worldwide [2]. However, to our knowledge, no studies evaluating the comorbidity of these two diseases have not yet been conducted. This study aimed to determinate the prevalence of sickle cell trait in people living with albinism in Kisangani (northeastern of Democratic Republic of the Congo).

## Methods

We conducted a cross-sectional descriptive survey in PLA attending the "Hôpital du Cinquantenaire de Kisangani" from the 30^th^ July to the 6^th^ September 2019, as well as among students in the Faculty of Medicine and Pharmacy at the University of Kisangani at the same period. In total, by non-probabilistic convenience sampling, we included 82 albinos and 139 non-albinos who constituted the control group. A total of 121 albinos visited the facility during the study period, but only 82 subjects who provided written consent or parental written consent for children, were included in the survey. Of all the students present at the time of the survey, 157 had willingness to provide written consent, but only 139 were included based on the inclusion criteria that were: not to be an albino and not having the history of albinism in the family. For haemoglobin phenotyping, blood samples were collected on "dried blood spot" (DBS) by capillary puncture and the analysis was performed by mass spectrometry at the Biochemical Genetics Laboratory of CHU of Liège. Data were entered into an Excel file and analyzed on SPSS 20.0 (Chicago, IL). In addition, the data were summarized in form of proportions and frequency tables for categorical variables. Continuous variables were summarized using means, median and standard deviation. P-value was computed for categorical variables using Chi-square (χ^2^) test. A p-value of less than 0.05 was considered to constitute a statistically significant difference.

## Results

Of the 221 subjects studied, 82 (37.1%) were albinos ranging in age from 1.6 to 48 years. Their mean age was 18.3±11.37 years and the median age was 17 years. The control group included 139 non albino subjects (62.9%) with no albinism family history. Their mean age was 26.9±8.19 years and the median age was 25.5 years. Forty-six (56.1%) of the 82 albinos were female; 43.9% male with a sex ratio of 1.28 (Table 1). Among albinos, 18.3% had AS hemoglobin (HbAS) and 81.7% hemoglobin AA (HbAA) compared to 18% of subjects with hemoglobin AS and 82% hemoglobin AA in the control group. The difference was not statistically significant (Chi-square=0.003, ddl=1, p=0.9544) (Table 2).

**Table 1:** Characteristics of the participants

Characteristics	F	M	Total	Mean Age±SD*(year)	Median age (year)	Extreme ages (year)
	Effectif(%)	Effectif (%)	Effectif(%)			
Albinos	46(56.1)	36(43.9)	82(100)	18.3±11.37	17	1.6 et 48
Non albinos	24(17.3)	115(82.7)	139(100)	26.9±8.19	25.5	3 et 62
Total	70(31.7)	151(68.3)	221(100)			

* Standard deviation

**Table 2:** Phenotype of hemoglobin

Characteristics	Hb AA	Hb AS	Total	p-value
	Effectif (%)	Effectif (%)	Effectif (%)	
Albinos	67(81.7)	15(18.3)	82(100)	0.9544
Non albinos	114(82)	25(18)	139(65.6)	
Total	181(81.9)	40(18.1)	221(100)	

Chi-square = 0.003, ddl=1

## Discussion

In this study, among albinos, females represented 56.1% (Table 1) with a female to male ratio of 1,28; 18.3% had hemoglobin AS (HbAS) and 81.7% hemoglobin AA(HbAA) compared to 18% of subjects with hemoglobin AS and 82% hemoglobin AA in the control group but with no statistically significant difference (p-value >0.05) (Table 2). Similarly to our results, Marçon CR *et al*. (2019), Emadi SE *et al*. (2017) and Yang *et al*. (2019) reported that most of the PLA were females, respectively 57.07%, 52.9% and 58.3% [9,19,20]. The same observation emerges from the studies of Massie RW and Hartmann RC (1957) and Aquaron R *et al*. (2007) [14,15]. In a most recent study conducted in Kinshasa on 200 albinos, Kakiesse V *et al*. reported the same finding with a sex ratio of 1.08 in favor of females [21]. However, the reason for this sex difference remains unclear. Hence, more studies are needed to elucidate this observation. Although albinism is an autosomal recessive genetic disease, such as sickle cell disease, at least for the oculocutaneous form, to date there is no formal evidence of a link between these two diseases.

Nevertheless, some observations suggested their possible association. Indeed, the first study that focused on the albinism and sickle cell disease association dates back to the 1950´s and was aroused by the observation of Massie RW and Hartmann RC in an African-American family with 4 albino children. One of them had the homozygous form of sickle cell disease and the other three had the sickle cell trait. Their study excluded an absolute linkage between sickle cell disease and albinism but claimed that a loose linkage was possible [14]. Later (2007), Aquaron R *et al*. reported, in the Ewondo ethnic group in Cameroon, a family of children presenting both albinism and sickle cell disease. In all three cases, it was oculocutaneous albinism type 2 (OCA2) with a homozygous 2.7-kb deletion of the P gene. In one case, oculocutaneous albinism type 2 (OCA2) was associated with the homozygous form of sickle cell disease and in the other two cases, OCA2 was associated with the sickle cell trait. However, the study remained silent on a possible link between the two diseases [15].

In a more recent study, on the basis of empirical observation that light-skinned sickle-cells patients tend to have less severe phenotypic expression of the disease when compared to those with darker skin color, Muideen *et al*. postulated the hypothesis that hypermelanotic or hypomelanotic skin status of homozygous SCD patient would influence the severity of the SCD phenotypic expression [1]. Our findings are therefore in agreement with that of Massie RW and Hartmann RC and indicates that the prevalence of sickle cell trait is not higher in albinos compared to non-albinos, since we did not observe a statistically significant difference between albinos and the control group (p=0,95). These results are not surprising as there is not any direct linkage between the albinism genes and the HbB gene. Moreover, our data suggest that the prevalence of sickle cell trait observed among albinos (18.3%) is similar to that expected in the general Congolese population. Indeed, Agasa B *et al*. reported the prevalence of 23.3% of the sickle cell trait in newborns in Kisangani (DRC) compared to about 1% for the homozygous form [17]. Similarly, Tshilolo *et al*. in Kinshasa observed a neonatal prevalence of sickle cell disease of 16.9% for the sickle cell trait and 1.4% for the homozygous form [16].

## Conclusion

Our results show that the prevalence of the sickle cell trait is high among people living with albinism, but does not differ from that observed in non-albinos in the Democratic Republic of the Congo. It is therefore important to raise awareness among people living with albinism about sickle cell disease and the importance of its premarital screening, as well as the general population. The relatively small number of albinos is a limitation of this study. The same applies to the selection of the control group made by convenience sampling. Nevertheless, this study contributes to the improvement of knowledge on the comorbidity sickle cell disease and albinism.

### What is known about this topic

- It has been suggested that there is no absolute linkage between sickle cell disease and albinism, but that a looser linkage was possible.

### What this study adds

- This study provides data on the comorbidity of sickle cell disease and albinism in sub-Saharan Africa;
- It also provides arguments that the prevalence of the sickle cell trait is not higher in people living with albinism, especially in the Democratic Republic of the Congo.
